# Flexible Electrochemical Biosensor Using Nanostructure-Modified Polymer Electrode for Detection of Viral Nucleic Acids

**DOI:** 10.3390/bios14120594

**Published:** 2024-12-04

**Authors:** Jiyu Han, Yejin Lee, Jin-Ho Lee, Jinho Yoon

**Affiliations:** 1 Department of Biomedical-Chemical Engineering, The Catholic University of Korea, Bucheon 14662, Republic of Korea; jiyu8852@catholic.ac.kr (J.H.); bioyjlee@catholic.ac.kr (Y.L.); 2 Department of Biotechnology, The Catholic University of Korea, Bucheon 14662, Republic of Korea; 3 School of Biomedical Convergence Engineering, Pusan National University, Yangsan 50612, Republic of Korea; leejh@pusan.ac.kr; 4 Department of Information Convergence Engineering, Pusan National University, Yangsan 50612, Republic of Korea

**Keywords:** flexible biosensor, nanotechnology, viral nucleic acids, electrochemistry, human papillomavirus

## Abstract

In the biosensor field, the accurate detection of contagious disease has become one of the most important research topics in the post-pandemic period. However, conventional contagious viral biosensors normally require chemical modifications to introduce the probe molecules to nucleic acids such as a redox indicator, fluorescent dye, or quencher for biosensing. To avoid this complex chemical modification, in this research, mismatched DNA with an intercalated metal ion complex (MIMIC) is employed as the probe sequence. In addition, the MIMIC is fabricated on a lithography-assisted nanostructure-modified flexible polymer electrode. On this flexible electrode, as a proof-of-concept study, a human papillomavirus (HPV-16 and -18) was detected by the MIMIC with a high accuracy. The developed biosensor exhibits an ultrasensitive ability to detect HPV in viral DNA without target amplification and chemical modifications in a simple preparation manner. Moreover, it retains its nanostructures and high conductivity after bending. In conclusion, the use of the proposed biosensor suggests a novel approach to developing an ultrasensitive and flexible biosensor for the detection of important biomarkers in a simple manner that can be applied in point-of-care testing.

## 1. Introduction

Recently, the accurate and early diagnosis of contagious viruses and diseases has become one of the most important issues in the field of biosensors [1,2]. In particular, this issue gained increased attention after the occurrence of the Corona virus disease in 2019 (COVID-19), which caused a global pandemic [3,4,5]. To achieve this, numerous studies have been conducted to develop a biosensor capable of the accurate diagnosis of viruses or diseases through the detection of target nucleic acids [6,7]. For most biosensors that have been reported, some techniques have been studied in detail, including fluorescent, electrochemical, optical, plasmonic, and Raman spectroscopic techniques [8,9,10,11]. However, in most cases, the additional introduction of probe molecules such as fluorescent dyes, redox-active molecules, and Raman-active molecules to nucleic acids is required to detect viruses or diseases using each technique [12,13], which inevitably leads to the need for a complex chemical modification process to introduce the probes to the nucleic acid at the nanometer scale. In addition, to achieve the ultrasensitivity required for a nucleic acid biosensor, prior to the detection of target nucleic acids, some pre-steps are normally required including a target amplification process like polymerase chain reaction (PCR) [14,15]. Even though some efficient amplification methods have been reported and used, such as the rolling circle amplification (RCA) method, these pre-amplification methods still require many additional components like primers, equipment, and sufficient operation time. The limitations of the introduction of additional probe molecules or pre-amplification steps hinder the achievement of nucleic acid detection in a simple and ultrasensitive manner for point-of-care testing applications [16,17]. Therefore, there is a clear need to develop nucleic acid biosensors that can be developed in a simple way while also enabling highly sensitive diagnoses.

Among the several techniques that are widely used in the development of nucleic acid biosensors, an electrochemical technique has some distinct advantages including a high sensitivity and selectivity, rapid response, and convenient investigation steps [18,19,20]. In addition, flexible or wearable biosensors have recently received much interest in the field of biosensors because they can be used for the real-time monitoring or diagnosis of disease, infections, and health conditions [21,22]. To develop flexible or wearable biosensors, an electrochemical technique has significant applicability because of its convenient operation with a flexible electrode [23,24]. Nevertheless, as mentioned above, the need for complex modification steps like the chemical modification of the redox-active molecules on the nucleic acids should be removed to develop an electrochemical biosensor capable of detecting viral nucleic acids using a simple detection method. As one strategy to address the chemical modification of redox-active molecules on the nucleic acids, mismatched nucleic acids and intercalation of metal ions in the mismatched nucleic acids can be utilized. There are reports that specific metal ions can be intercalated into certain mismatched sequences to stabilize the mismatched sequence through the formation of covalent bonding between the mismatched nucleic acid and metal ion [25,26,27]. For instance, when a cytosine–cytosine (C-C) mismatched sequence exists in double-stranded DNA (dsDNA), silver (Ag) ions can intercalate into the C-C mismatched sequence [28]. In addition, this intercalation process is performed spontaneously like self-assembly without any external modification steps. Furthermore, intercalated metal ions exhibit inherent redox signals which are bigger than those of the conventionally used redox-active molecules such as anthraquinone and ferrocene. Therefore, through the introduction of a phenomenon composed of mismatched nucleic acids and the intercalation of metal ions and a conductive flexible electrode, a flexible electrochemical biosensor can be developed to detect the viral nucleic acids present in the sample in a highly sensitive manner of detection. 

**Figure 1:**
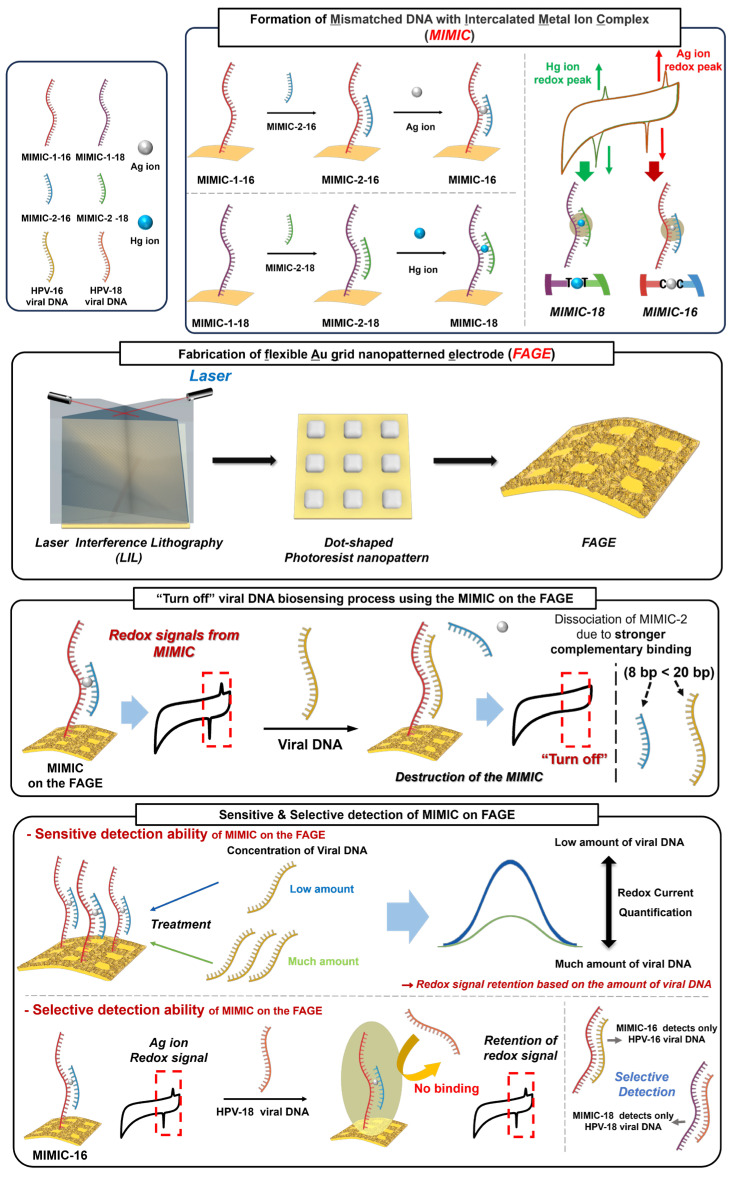
Schematic illustration of flexible electrochemical biosensor composed of MIMIC and FAGE for detection of viral DNAs.

In this study, a flexible electrochemical biosensor composed of **m**ismatched DNA with an **i**ntercalated **m**etal **i**on **c**omplex (MIMIC) on a lithography-assisted nanostructure-modified flexible polymer electrode is developed for the detection of viral nucleic acid (Figure 1). MIMIC composed of MIMIC-1 and MIMIC-2 is employed as a redox-active probe capable of capturing viral nucleic acid. After the hybridization of MIMIC-1 and MIMIC-2, they can form a C-C mismatched sequence for Ag ion intercalation and a thymine–thymine (T-T) mismatched sequence for mercury (Hg ion) intercalation, respectively. Using the thiol group at the end of MIMIC-1, this MIMIC can be immobilized directly on a gold (Au) surface. In the presence of a viral nucleic acid sequence, short MIMIC-2 is de-hybridized from the MIMIC due to the high binding affinity between the viral nucleic acid and its complementary sequence (MIMIC-1). After the viral nucleic acid has been captured, newly prepared dsDNA has no mismatched sequence, so no more Ag ions can exist on the electrode. Therefore, by analyzing the redox signals of the intercalated Ag ions, the presence of the viral nucleic acid can be determined. In addition, to enhance the sensitivity of this flexible biosensor, we fabricated a lithography-assisted **f**lexible **A**u **g**rid nanopatterned **e**lectrode (FAGE) using a laser interference lithography (LIL) technique on the gold-coated flexible polymer electrode. By utilizing the LIL technique, a homogeneous metallic nanostructure can be fabricated on the conductive polymer electrode, which allows us to achieve an effective electron transfer and rapid response due to there being a high surface area and excellent conductivity [29,30]. As a proof-of-concept study, human papillomavirus 16 (HPV-16) and 18 (HPV-18) were selected as the target viral DNAs as they can induce the occurrence of cervical cancer, so the early diagnosis of HPV-16 is important [31,32]. In short, by combining MIMIC and FAGE, a highly sensitive flexible electrochemical can be developed for the detection of HPV-16 and -18. In addition, this flexible biosensor can be utilized for multiple or selective diagnoses of HPV-16 and HPV-18 by using two different MIMICs composed of C-C mismatch/Ag ions and T-T mismatch/Hg ions, respectively.

## 2. Materials and Methods

### 2.1. Materials

A gold-coated silicon electrode, which consisted of gold (50 nm), Cr (2 nm) on the silicon dioxide (SiO_2_) wafer, was purchased from the National Nanofab Center (Daejeon, South Korea). A gold-coated flexible polymer electrode composed of gold on polyimide film was obtained from GMEK Technologies (Anyang, South Korea). The photoresist (PR) solvent (AZ EBR solvent), UV-cross-linkable PR (AZ2020), and developer solution (AZ 300 MIF developer) were procured from Merck KGaA (Darmstadt, Germany). Additionally, dimethyl sulfoxide (DMSO) was acquired from Corning (Corning, New York, NY, USA). Sulfuric acid and hydrogen peroxide for piranha solution were purchased from Daejung chemical (Siheung, South Korea). Sylgard 184 Silicone elastomer base and a curing agent for Polydimethylsiloxane (PDMS) were purchased from Dowhitech (Seongnam, South Korea). All ssDNAs used in this study including thiolated MIMIC-1-16 (5′-TGAAGTAGATATGGCAGCACGAGAGAATGG-thiol-3′), thiolated MIMIC-1-18 (5′-ACAATATGTGCTTCTACTCAAAAAG-thiol-3′), MIMIC-2-16 (5′-GCTCCCATA-3′), MIMIC-2-18 (5′-TAGTAGCAC-3′), HPV-16 viral DNA (5′-GTGCTGCCATATCTACTTCA-3′), and HPV-18 viral DNA (5′-TGTGTAGAAGCACATATTGT-3′) were synthesized and ordered from Bioneer (Daejeon, South Korea). Diethyl pyrocarbonate (DEPC) water for DNA dilution and a gold plating solution were purchased from Thermo Fisher Scientific, (Waltham, MA, USA). The silver nitrate (AgNO_3_) and ethanol were purchased from Daejung chemical (Siheung, South Korea). The mercury chloride (HgCl_2_) was purchased from Sigma-Aldrich (Saint Louis, MO, USA). The phosphate-buffered saline (PBS) solution was prepared using PBS powder obtained from Sigma-Aldrich (Saint Louis, MO, USA). Deionized water (DIW) was prepared by using the Wellix Plus set (Jieo tech, Cheongju, South Korea). In addition, Triton X-100 was ordered from Sigma-Aldrich (USA) to clean the surface of the flexible polymer electrode. Pipette tips were purchased from Sigma-Aldrich (USA), which were used as the chamber attached to the electrode. Potassium hexacyanoferrate (II) trihydrate and hexacyanoferrate (III) were purchased from Sigma-Aldrich (USA), which were used for an investigation into enhancing the electrochemical signals of the prepared electrode.

### 2.2. Fabrication of FAGE

First, the gold-coated flexible polymer electrode was cleaned with 1% Triton X-100 for 20 min under sonication, followed by an ethanol wash under the same conditions. Next, an AZ2020 photoresist solution, diluted to a ratio of 6:4 with AZ EBR solvent, was spin-coated onto the gold-coated flexible polymer electrode by using 20 µL drops and spinning at 4000 rpm for 20 s. After the formation of the PR layer on the electrode by spin coating, it was prebaked on a hot plate at 95 °C for 60 s before being exposed to UV light (λ = 325 nm, 1.2 mW) using a He-Cd laser (Kimmon Koha Laser Systems, Tokyo, Japan). During this laser exposure process, the PR was selectively cured due to the amplification effect of light directly irradiating the substrate from the light source and light reflected from the Lloyd’s mirror onto the substrate, which allowed for the formation of a regular PR nanopattern on the electrode. Based on our previous results, an incident angle of 5.82° was used to create a PR pattern with a 1600 nm pitch size. To generate a dot-shaped PR nanopattern, the PR spin-coated electrode was exposed to UV light once, rotated 90°, and then exposed once more for 10 s each time. Afterward, a post-bake was performed at 105 °C for 60 s to stabilize the cross-linked PR nanopatterns. To remove the uncured PR and leave only the dot-shaped PR nanopatterns, the electrode was immersed in a developer solution for 160 s, ultimately producing a polymer electrode with regularly arranged dot-shaped PR nanopatterns of 800 nm in size.

To make the FAGE, first, the tip of a 1000 µL pipette was cut off and cleaned with ethanol to be used as a chamber. Next, it was attached to the electrode composed of dot-shaped PR nanopatterns using PDMS. After the attachment of the tip using PDMS, it was placed on an 85 °C hot plate for 40 min to ensure proper adhesion of the chamber to the electrode through the hardening of the PDMS. After this step, electrochemical deposition was carried out using a conventional three-electrode system, consisting of a platinum wire as the counter electrode, a silver/silver chloride (Ag/AgCl) double-junction electrode as the reference electrode, and the electrode composed of the dot-shaped PR nanopatterns as the working electrode. During the electrochemical deposition, 300 µL of gold plating solution was stored inside the chamber, and the electrochemical deposition of gold was performed by applying the amperometric i-t curve technique for 200 s using the CHI 601E potentiostat workstation (CHInstruments, Austin, TX, USA). The detailed operation conditions were an applied potential of -0.95 V, a sampling interval of 0.1 s, a quiet time of 2 s, and a sensitivity of 1.0 × 10^−4^ (A/V). After the electrochemical deposition of gold onto the region of the electrode in which the dot-shaped PR nanopatterns were not placed, the electrode was treated with DMSO for 15 min to remove the dot-shaped PR nanopatterns and retain only the electrodeposited gold. After washing with DIW, the FAGE was finally fabricated.

### 2.3. Preparation of MIMIC on the FAGE and Its Biosensing Mechanism

To detect HPV viral DNA, first, the MIMIC was fabricated on the FAGE. Two different MIMICs (MIMIC-16 and MIMIC-18) were used in this study. The MIMIC-16 was composed of dsDNA with C-C mismatch and intercalated Ag ions, and the MIMIC-18 was composed of dsDNA with T-T mismatch and intercalated Hg ions. Specifically, to make the MIMIC-16 capable of HPV-16 detection, MIMIC-1-16 and MIMIC-2-16 were prepared on the FAGE with Ag ion intercalation. To make the MIMIC-18 capable of HPV-18 detection, MIMIC-1-18 and MIMIC-2-18 were prepared on the FAGE with Hg ion intercalation. 

To fabricate the MIMIC-16 on the FAGE, the FAGE was first washed with DIW. On the cleaned FAGE, 5 μL of 2.5 μM MIMIC-1-16 dissolved in DEPC was immobilized for 2 h at 4 °C via the formation of a self-assembled monolayer between the thiol group of the MIMIC-1-16 and gold surface of the FAGE. Next, 10 μL of 2.5 μM MIMIC-2-16 was immobilized on the MIMIC-1-16-modified FAGE for 2 h at 4 °C. After the formation of dsDNA composed of MIMIC-1-16 and MIMIC-2-16, one C-C mismatched sequence was formed inside the dsDNA. After that, 100 μL of 50 μM AgNO_3_ dissolved in DIW was added to the dsDNA-immobilized FAGE for 1 h at 4 °C to intercalate Ag ions inside the C-C-mismatched sequence. After being washed with DIW, MIMIC-16 was finally prepared on the FAGE. To fabricate the MIMIC-18 on the FAGE, all of the fabrication steps were exactly same as the fabrication steps used for MIMIC-16, but MIMIC-1-18, MIMIC-2-18, and Hg ions were used instead of MIMIC-1-16, MIMIC-2-16, and Ag ions.

The biosensing mechanism of detecting HPV-16 viral DNA using MIMIC-16 on the FAGE is provided below. HPV-16 viral DNA has a greater number of base pairs complementary to MIMIC-1-16 that are directly attached to the FAGE compared to MIMIC-2-16, leading to the replacement of the hybridized sequence for the MIMIC-1-16 with that for MIMIC-2-16 on the HPV-16 viral DNA. Thus, the MIMIC-16 with intercalated Ag ions was destroyed and the newly formed dsDNA composed of the MIMIC-1-16 and HPV-16 viral DNA no longer contained the mismatched sites, so the Ag ions were released from the dsDNA. Therefore, in the absence of HPV-16 viral DNA, the redox signals of the Ag ions intercalated in the MIMIC-16 were measured electrochemically, but in the presence of HPV-16 viral DNA, the signals from the Ag ions disappeared. Through the “Turn-off” type biosensing process, the detection of the HPV-16 viral DNA can be carried out by using the MIMIC-16 on the FAGE. In the case of HPV-18 viral DNA detection, the biosensing mechanism is the same as above, but instead of MIMIC-16 with Ag ions, MIMIC-18 with Hg ions was used as the probe for HPV-18 viral DNA detection. Therefore, in the case of HPV-18 detection, redox signals from the Hg ions were measured in the absence of HPV-18 viral DNA, but these signals from the Hg ions disappeared in the presence of HPV-18 viral DNA.

### 2.4. Electrochemical Detection of Viral DNAs Using MIMIC on the FAGE

By using the biosensing mechanism presented in the above section, the electrochemical detection of HPV-16 and HPV-18 was conducted electrochemically. First, the redox signals of the MIMIC on the FAGE were investigated using the cyclic voltammetry (CV) technique in the CHI 601E potentiostat workstation to obtain the redox signals from the Ag and Hg ions, respectively. During the electrochemical investigation, PBS was used as the electrolyte and a three-electrode system composed of a platinum wire electrode (The counter electrode), a Ag/AgCl double-junction electrode (The reference electrode), and the MIMIC on the FAGE (The working electrode) was utilized. As the CV parameters, a potential range of 0.6 to −0.1 V for the MIMIC-16 and 0.6 to −0.5 V for MIMIC-18, with a sample interval of 0.001 V, a scan rate of 0.05 V/s, and a quiet time of 2 s, were applied. 

After the measurement of the redox signals from the MIMIC-16 and MIMIC-18 on the FAGE, HPV-16 and -18 viral DNAs were treated with the MIMIC-16 and MIMIC-18 on the FAGE, respectively, for 2 h at 4 °C. The concentration of HPV viral DNA treated ranged from 1 μM to 1 pM. Then, to evaluate the detection of HPV viral DNA using the disappearance of the redox signals of the MIMIC on the FAGE, an electrochemical investigation was conducted using the same conditions as above.

## 3. Experimental Results

### 3.1. Confirmation of FAGE Fabrication

The surfaces of the FAGE and PR pattern were characterized using scanning electron microscopy (SEM, S-4800, HITACHI, Japan) and atomic force microscopy (AFM, MULTIMODE-8-AM, Bruker, Karlsruhe, Germany). For the SEM analysis of the PR pattern, a platinum was sputtered on the PR pattern to create a conductive surface. For the AFM analysis, tapping-mode AFM was used to investigate the nanometer-scale images of the FAGE. Additionally, surface roughness and height profile analyses were performed, and 3D-converted images were obtained. To validate the enhancement of the redox signal using the FAGE, potassium hexacyanoferrate (II) trihydrate and hexacyanoferrate (III) were used as electrolytes. The CV technique was utilized to investigate the redox signals from the electrolytes on both the FAGE and the gold-coated flexible silicon electrode. During the CV investigation, a potential range of −0.2 to 0.6 V, with a scan rate of 0.1 V/s, a sample interval of 0.001 V, a quiet time of 2 s, and a sensitivity setting of 1.0 × 10^−4^ A/V were used as parameters.

**Figure 2:**
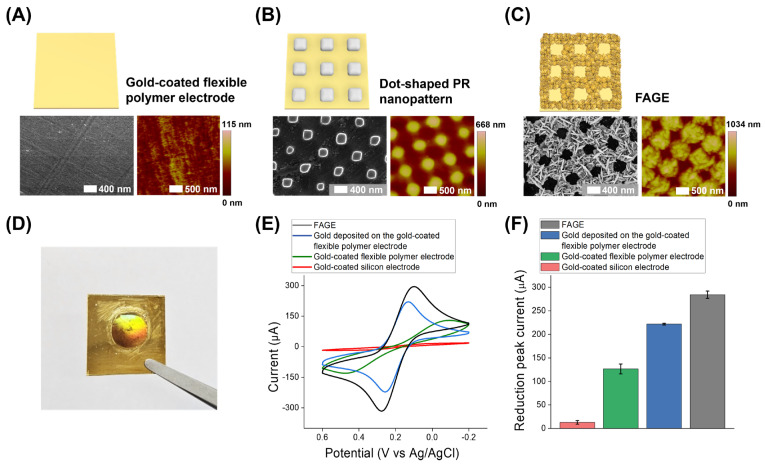
Confirmation of FAGE fabrication. Schematic image of and SEM and AFM results for (**A**) a gold-coated flexible polymer electrode, (**B**) a dot-shaped PR nanopattern and (**C**) FAGE. (**D**) An optical image of the FAGE displaying holographic properties; (**E**) cyclic voltammograms of potassium hexacyanoferrate (II) trihydrate and hexacyanoferrate (III) on the FAGE, the bare gold-coated flexible polymer electrode, the electrochemically deposited gold on the gold-coated flexible polymer electrode without photoresist, and the gold-coated silicon electrode; and (**F**) the average reduction peak current values of potassium hexacyanoferrate (II) trihydrate and hexacyanoferrate (III) on the FAGE, the bare gold-coated flexible polymer electrode, the electrochemically deposited gold on the gold-coated flexible polymer electrode without photoresist, and the gold-coated silicon electrode. Error bars exhibit the standard deviations of three different measurements.

The results of the SEM and AFM analyses presented in Figure 2 display the surface characteristics of the gold-coated flexible polymer electrode at each fabrication step, leading to the formation of the FAGE. In Figure 2A, the SEM image reveals the surface morphology of the gold-coated flexible polymer electrode, which serves as the base substrate. It showed regularly deposited gold grains on the electrode that were around 15 nm in height (Figure S2), as well as a scratch which may have been produced during the preparation of the electrode by the company (GMEK Technologies, Anyang, South Korea). After PR pattern fabrication on the gold-coated flexible polymer electrode, as shown in Figure 2B, the dot-shaped PR nanopatterns were fabricated uniformly and regularly on the gold-coated flexible polymer electrode. Each dot-shaped PR nanopattern showed an approximately 800 nm width and a 350 nm height, as confirmed by the SEM and AFM measurements. Figure 2C displays the surface morphology of the FAGE, which consists of a grid-shaped gold nanostructure, which shows around an 800 nm width and a 300 nm height for the electrochemically deposited gold. The fabrication of the FAGE was also confirmed by the hologram observed in the FAGE-fabricated circular area in the optical image of the electrode (Figure 2D), which emerged from the reflection of light off the periodic nanostructures on the electrode, indicating the successful formation of the FAGE. Through the sectional analysis (Figure S2), it was confirmed that the surface roughness of the FAGE had increased compared to the gold-coated flexible polymer electrode, and this rougher surface can extend the surface area available for efficient MIMIC immobilization. Consequently, this lead to an improved conductivity and enhanced sensitivity of the developed biosensor through the facilitation of the electron transfer of redox molecules.

Next, to verify the enhanced conductivity of the FAGE based on the extended area of gold grid nanopattern formation, a CV investigation was conducted using potassium hexacyanoferrate (II) trihydrate and hexacyanoferrate (III) prepared at 1:1 ratio with electrolytes. As shown in Figure 2E, the FAGE exhibited around 15-times-enhanced redox signals with a reduction peak current of 295.2 μA compared to that of the gold-coated silicon electrode (with a reduction peak current of 18.9 μA). It also showed reproducible results with an average reduction peak current of 284.3 μA compared to the gold-coated silicon electrode (with an average reduction peak current of 13.1 μA) with approximately 21 times signal enhancement (Figure 2F). Furthermore, to verify the electrochemical signal enhancement effect of the FAGE more clearly, a bare gold-coated flexible polymer electrode and the electrochemically deposited gold on a gold-coated flexible polymer electrode without photoresist were prepared and used for the same electrochemical investigation. As shown in the other cyclic voltammograms and bar graphs in Figure 2E,F, the reduction peak currents of the bare gold-coated flexible polymer electrode and the electrochemically deposited gold on the gold-coated flexible polymer electrode without photoresist were obtained at 129.2 μA and 220.4 μA, respectively (the average reduction peak currents are 126.6 μA and 221.8 μA, respectively). Since the side areas of the gold nanostructures of the FAGE were also used for electrochemical investigation, an extension of the conductive surface area was achieved that induced the enhancement of conductivity compared to the flexible electrode without nanostructures and the electrode prepared using the same electrochemical deposition of the gold but without photoresist. In addition, through a zoomed-out analysis using SEM, it could be seen that a large area with homogeneously prepared nanostructures was fabricated well (Figure S1). These results proved that the FAGE was successfully developed, and its conductive properties are suitable for ultrasensitive electrochemical biosensing, while retaining its flexibility.

### 3.2. Flexibility of FAGE

**Figure 3:**
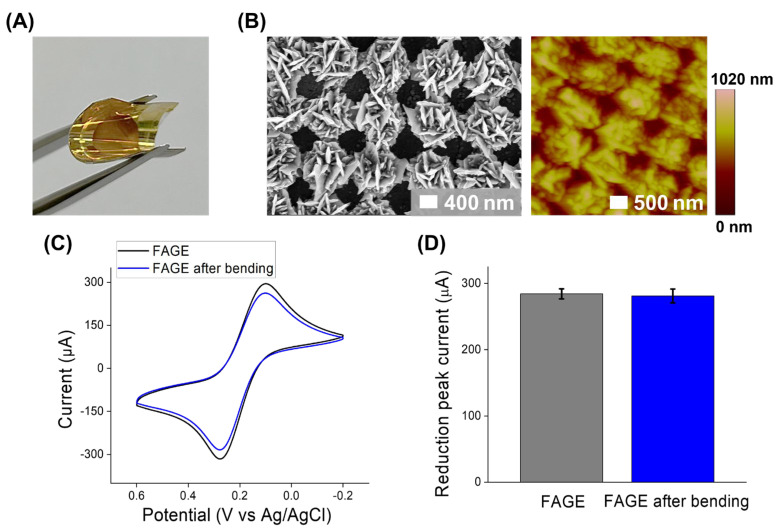
Flexibility of the FAGE. (**A**) The optical image of the bent FAGE; (**B**) surface morphological analysis of the FAGE after bending using SEM and AFM.; (**C**) cyclic voltammograms of potassium hexacyanoferrate (II) trihydrate and hexacyanoferrate (III) on the FAGE before and after bending; and (**D**) the average reduction in the peak current values of potassium hexacyanoferrate (II) trihydrate and hexacyanoferrate (III) on the FAGE before and after bending. Error bars exhibit the standard deviations of three different measurements.

The flexibility of the fabricated FAGE was evaluated after multiple bending processes. As shown in Figure 3A, we bent the developed FAGE manually, repeated the process several times, and stored it in a bent state. During the bending process, the FAGE exhibited its holographic properties well due to the non-destruction of uniform and regular nanostructures. After this severe treatment, we investigated the FAGE to verify its flexibility to be used in the development of a flexible biosensor. Figure 3B shows the SEM analysis results for the FAGE after the bending process. The surface morphology of the FAGE maintained its unique grid-shaped gold nanostructure without any cracks, which shows width of around 800 nm, which is most similar to its original structure shown in Figure 2 before the bending process. Through additional AFM analysis, we also found that the FAGE retained its height of around 300 nm, similar to the height obtained before the bending (Figure S2D).

Next, to confirm that the FAGE maintains its excellent conductivity even after the bending process, we carried out an investigation similar to the electrochemical investigation performed on the FAGE before bending. To achieve this, a CV investigation was conducted using potassium hexacyanoferrate (II) trihydrate and hexacyanoferrate (III) prepared using a 1:1 ratio with electrolytes. As shown in Figure 3C, after multiple bending process, the FAGE exhibited an excellent conductivity with a reduction peak current of 262.5 μA, which was only reduced by 1.1% compared to the reduction peak current value of the FAGE before bending (the reduction peak current before bending was 295.2 μA). It also showed that the flexibility shown in Figure 3D was reproducible after repetitive measurements using different FAGEs (the average reduction peak currents before and after the bending were 284.3 μA and 281.2 μA, respectively). Furthermore, to investigate the retention of its conductive properties during the bent state, as shown in Figure S3, the FAGE was attached to a curved surface and used for an electrochemical investigation. For this, a CV investigation was conducted using potassium hexacyanoferrate (II) trihydrate and hexacyanoferrate (III) under the same experimental conditions used in the above experiment. During the bent state, the FAGE exhibited a reduction peak current of 274.1 μA (the average reduction peak current was 267 μA), which was similar to the results from the FAGE before and after bending. These results verified that the FAGE has a sufficient flexibility to be used as an electrode for development of flexible biosensors.

### 3.3. Electrochemical Investigation of the MIMIC Prepared on the FAGE

**Figure 4:**
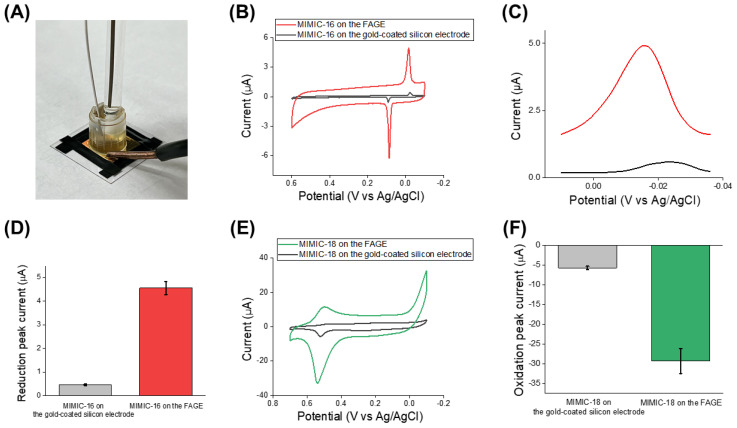
Electrochemical investigation of the MIMIC. (**A**) Optical image of CV investigation of the MIMIC on the FAGE; (**B**) cyclic voltammograms of MIMIC-16 on the FAGE and gold-coated silicon electrode; (**C**) zoomed-in cyclic voltammogram; and (**D**) its reproducibility results. (**E**) Cyclic voltammograms of MIMIC-18 on the FAGE and gold-coated silicon electrode, and (**F**) its reproducibility results. All error bars exhibit the standard deviations of four different measurements.

To investigate the electrochemical properties of the MIMIC-16 fabricated on the FAGE, a CV investigation was conducted under the same operation conditions as used above. Also, to verify the redox signal enhancement of the MIMIC-16 by the highly conductive FAGE, as a control, MIMIC-16 was prepared on the conventional gold-coated silicon electrode. To make a precise comparison, the immobilization area of the MIMIC-16 was the same for both types of electrodes using the cleaned cut-off 1000 µL pipette tip as a chamber, as shown in Figure 4A. Figure 4B shows the cyclic voltammograms of the redox signals derived from the MIMIC-16. Comparing the reduction peak current value in each case, the reduction peak current value of the MIMIC-16 on the FAGE showed a value of 4.92 μA, which was around 10 times higher than that of the MIMIC-16 on the gold-coated silicon electrode (the reduction peak current value was 0.41 μA). The enhancement in the redox signal can be seen more clearly in the zoomed-in cyclic voltammogram in Figure 4C. A small peak shift was observed that was presumed to be the result of the gold clusters in the deposited FAGE affecting the electron transfer of Ag ions from the MIMIC-16. From results, in addition to the verification of the enhancement of the redox signal of the MIMIC-16 by the FAGE, we verified that the MIMIC-16 was successfully developed on the FAGE, because the acquired redox peak signals derived from the Ag ions in the MIMIC-16 were mostly similar to the redox peak locations of Ag ions reported in other studies [6,28]. It also showed reproducible results with 4.56 μA for the FAGE and 0.46 μA for the gold-coated silicon electrode (Figure 4D). 

In the case of the MIMIC-18-immobilized FAGE, as in the case of MIMIC-16, excellent redox signals were measured with the oxidation peak current value of −32.8 μA at around 0.55 V potential, which was derived from Hg ions intercalated in the MIMIC-18. This was much higher than that obtained from the MIMIC-18 on the gold-coated silicon electrode (the oxidation peak current value was −5.74 μA) (Figure 4E). Since the oxidation peak current was more sensitive in the case of the MIMIC-18, we used the oxidation peak current, instead of the reduction peak current, to analyze the redox signals. In addition, the MIMIC-18 on the FAGE showed a sufficient reproducibility with the oxidation peak current value of −29.3 μA for the FAGE, which was much higher than that of the MIMIC-18 on the gold-coated silicon electrode with the oxidation peak current value of −5.74 μA (Figure 4F). From the results, we verified that the MIMIC-16 and MIMIC-18 were fabricated on the FAGE with highly enhanced signals and reproducibility, which could be used for the biosensing of HPV-16 and -18 viral DNA. 

### 3.4. Results for Electrochemical Detection of Viral DNAs Using MIMIC on the FAGE

**Figure 5:**
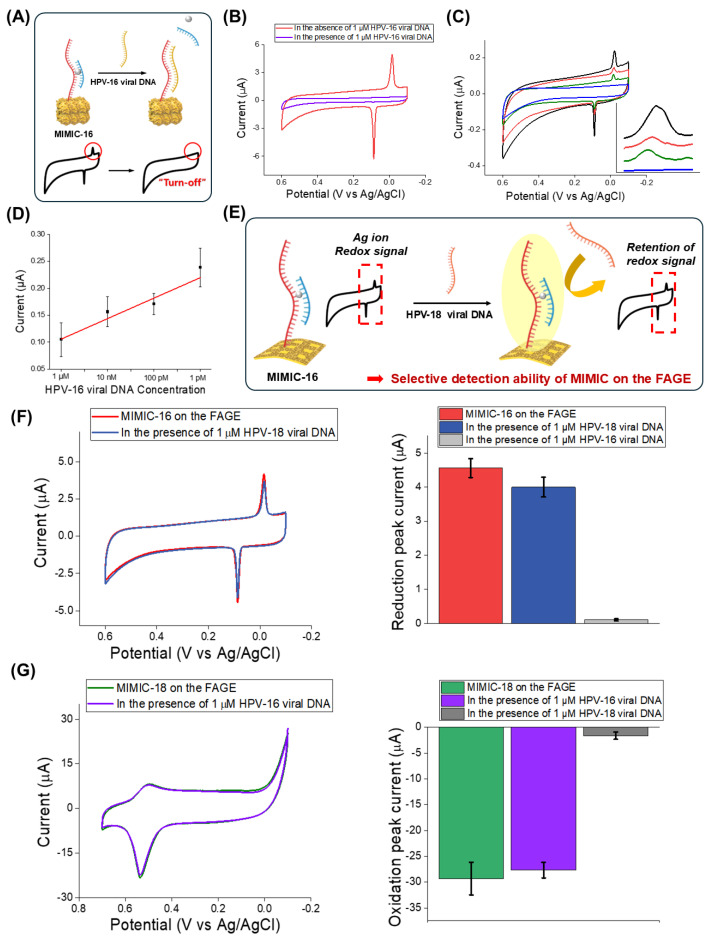
Electrochemical detection of viral DNAs using MIMIC on the FAGE. (**A**) Scheme for biosensing mechanism of the MIMIC on the FAGE; (**B**) cyclic voltammograms of the MIMIC-16 on the FAGE in the absence or presence of HPV-16 viral DNA; (**C**) cyclic voltammograms and zoomed-in reduction current peaks of the MIMIC-16 on the FAGE in the presence of different amounts of the HPV-16 viral DNA (From 1 μM to 1 pM); (**D**) linear response upon addition of HPV-16 viral DNA from 1 μM to 1 pM; (**E**) scheme for selective biosensing mechanism, cyclic voltammograms, and reproducibility results of (**F**) MIMIC-16 and (**G**) MIMIC-18 in the presence of HPV-18 or -16 viral DNA. All error bars exhibit the standard deviations of four different measurements.

To investigate the biosensing ability of the MIMIC on the FAGE for the detection of HPV viral DNAs, first of all, the 1 μM HPV-16 viral DNA was treated on the MIMIC-16 prepared FAGE. After the HPV-16 viral DNA had been captured by the MIMIC-16 and washed with DIW, an electrochemical investigation was conducted using the CV technique. As we expected (Figure 5A), in the presence of the 1 μM HPV-16 viral DNA, as shown in Figure 5B, the measured reduction peak current completely disappeared compared to that of the MIMIC-16 on the FAGE in the absence of the 1 μM HPV-16 viral DNA. Based on the obtained result, we reduced the amount of the HPV-16 viral DNA added to the MIMIC-16 on the FAGE. Figure 5C shows the cyclic voltammograms and zoomed-in reduction current peaks for the MIMIC-16 on the FAGE in the presence of different amounts of HPV-16 viral DNA (from 1 μM to 1 pM). The number of MIMIC-16 dissociations and the number of Ag ions released were determined according to the amount of HPV-16 viral DNA present, and as the amount of HPV-16 viral DNA present decreased, the reduction in the peak current increased proportionally. After several identical biosensing investigations, we found a linear response with a high reproducibility, as shown in Figure 5D (R^2^ = 0.9), for the detection of HPV-16 viral DNA using MIMIC-16 on the FAGE. In the case of the MIMIC-18 on the FAGE, it detected the HPV-18 viral DNA well, similar to the case using MIMIC-16 for the detection of the HPV-16 viral DNA (Figure S5).

Next, to investigate the selective detection ability of this biosensor, we conducted cross-tests between MIMIC-16 and MIMIC-18 on the FAGE using HPV-16 and -18 viral DNAs. For this, HPV-18 viral DNA was treated to the MIMIC-16 on the FAGE and HPV-16 viral DNA was treated to the MIMIC-18 on the FAGE, respectively (Figure 5E). As shown in Figure 5F, unlike when treating HPV-16 viral DNA, there was only a small decrease in redox signal of the MIMIC-16 in the presence of HPV-18 viral DNA, which verified that MIMIC-16 detects only HPV-16 viral DNA in a highly selective manner (in the absence and presence of HPV-18 viral DNA on the MIMIC-16, the reduction peak current values were 3.68 μA and 4.18 μA , respectively). Similar to this result, in the presence of HPV-16 viral DNA, the MIMIC-18 also retained its redox signal well (in the absence and presence of HPV-16 viral DNA on the MIMIC-18, the oxidation current values were −22.4 μA and −23.3 μA , respectively), which verified the selective biosensing ability of the MIMIC-18 on the FAGE for only HPV-18 viral DNA (Figure 5G). In addition, even though a small decrease in the peak current values was detected, which may have been due to the washing step after the viral DNA treatment, they showed reproducible results. In the case of the MIMIC-16 on the FAGE, its original reduction peak current value was 4.56 μA, and this was 4 μA and 0.11 μA in the presence of HPV-18 and -16 viral DNAs on the MIMIC-16, respectively. Also, In the case of the MIMIC-18 on the FAGE, its original oxidation peak current value was −29.3 μA, and this was −27.7 μA and −1.61 μA in the presence of HPV-16 and -18 viral DNAs on the MIMIC-18, respectively. In addition to the cross test using HPV-16 and -18 viral DNAs, another control DNA sequence (5′-TATTTTCCCTTT-3′) and some chemicals including hydrogen peroxide and glucose were applied to the MIMIC-16- and MIMIC-18-modified FAGE. In the case of the MIMIC-16-modified FAGE, in the presence of the control DNA, hydrogen peroxide, and glucose, the MIMIC-16 maintained its reduction signals well due to the non-destruction of the MIMIC-16 structure. This result was also obtained in the case of the MIMIC-18 when the control DNA and chemicals were applied (Figure S6). These results supported that our developed flexible biosensor composed of the MIMIC and the FAGE has highly sensitive and selective detection properties for viral DNAs, retaining its flexibility.

## 4. Conclusions

In the field of biosensors, the development of flexible/wearable biosensors capable of contagious virus detection has received huge attention for point-of-care testing applications in recent years. However, to develop these biosensors, sophisticated pre-steps such as PCR and the chemical modification of probe molecules like fluorophore and Raman reporters on the nucleic acids should be removed. To address these issues, in this study, MIMIC composed of mismatched nucleic acids and intercalated metal ions was developed as a biosensing probe attached to a highly conductive and flexible FAGE for the detection of HPV-16 and -18 viral DNAs in a simple manner. The flexible biosensor developed successfully detected the HPV-16 viral DNA (1 pM detection limit) without PCR and chemical modification steps. In addition, it retained its nanopatterned FAGE structure and biosensing ability well after several bending processes. Furthermore, based on the developed MIMICs (MIMIC-16 or MIMIC-18) applied to the FAGE, HPV-16 or HPV-18 viral DNAs were selectively detected through the measurement of redox signals from Ag or Hg ions. Information about the DNA sequences used in this study is provided in Figure S4. Despite the excellent conductivity and flexibility of the FAGE for biosensing applications, a practical wearable biosensing application using a real sample was not demonstrated here because of the target’s restrictions (viral DNA). However, by using the FAGE as an electrode, a practical wearable biosensing system can be developed through the introduction of other molecule-targeting probes such as aptamers to broaden target ranges. In addition, since some toxic ions such as Ag or Hg ions are required for the development of MIMIC, some restrictions exist in terms of directly biosensing living cells, but it can be used for extracellular biosensing applications with a high accuracy. Also, even though a multiplex detection was not demonstrated here, we anticipate that follow-up studies may be able to achieve this by optimizing the number of metal ions intercalated into the MIMICs and the simultaneous introduction of different types of MIMICs into the same FAGE. In addition, by optimizing the MIMIC structure with optimal numbers of intercalated metal ions, the sensitivity of the biosensor can be improved. Nevertheless, in this study, we demonstrated the simple biosensing of viral DNAs using MIMIC and flexible FAGE for the first time. In conclusion, the flexible biosensor developed composed of the MIMIC on the FAGE can provide a novel strategy to develop an ultrasensitive and flexible biosensor for the detection of diverse important biomarkers in a simple detection manner that can be applied in point-of-care testing.

## Data Availability

All data are provided in the manuscript.

## Supplementary Materials

The following supporting information can be downloaded at: https://www.mdpi.com/article/10.3390/bios14120594/s1, Figure S1: Zoomed-out SEM results of (A) -coated flexible polymer electrode, (B) -shaped PR nanopatterns, (C) FAGE, and (D) FAGE after bending. Figure S2: 3D-converted AFM images with vertical investigation of (A) -coated flexible polymer electrode, (B) -shaped PR nanopatterns, (C) FAGE, and (D) FAGE after bending. Figure S3: (A) The optical image of the bent FAGE during the electrochemical investigation, =; (B) voltammograms of potassium hexacyanoferrate (II) trihydrate and hexacyanoferrate (III) on the bent FAGE; and (C) the average reduction peak current values of potassium hexacyanoferrate (II) trihydrate and hexacyanoferrate (III) on the bent FAGE and the FAGEs before and after bending. Error bars exhibit the standard deviations of three different measurements. Figure S4: Information about DNA sequences used in the development of the flexible electrochemical biosensor composed of MIMIC on the FAGE, and target viral nucleic acids. Figure S5: Cyclic voltammograms of MIMIC-18 on the FAGE and that in the presence of 1 μM HPV-18 viral DNA. Figure S6: The average redox peak current values of the (A) MIMIC-16 and (B) MIMIC-18 during the addition of different molecules (control DNA, hydrogen peroxide, and glucose). Error bars exhibit the standard deviations of three different measurements.
