# Supplementary material for: Flexible Electrochemical Biosensor Using Nanostructure-Modified Polymer Electrode for Detection of Viral Nucleic Acids
Source: Biosensors (Basel). 2024 Dec 4;14(12):594. doi: 10.3390/bios14120594 (PMC11674301; doi:10.3390/bios14120594)
Supplement: Supplementary file 1 [file biosensors-14-00594-s001.zip › biosensors-3309365-supplementary.pdf]

biosensors

MDPI

Supplementary materials

Flexible electrochemical biosensor using nanostructure modified polymer electrode for detection of viral nucleic acids

Jiyu Han[1,†], Yejin Lee [1,2,†], Jin-Ho Lee[3,4] and Jinho Yoon [1,2,*]

1 Department of Biomedical-Chemical Engineering, The Catholic University of Korea, Bucheon, 14662, Republic of Korea; jiyu8852@catholic.ac.kr, bioyjlee@catholic.ac.kr, jyoon@catholic.ac.kr
2 Department of Biotechnology, The Catholic University of Korea, Bucheon, 14662, Republic of Korea; bioyjlee@catholic.ac.kr, jyoon@catholic.ac.kr
3 School of Biomedical Convergence Engineering, Pusan National University, Yangsan, 50612, Republic of Korea; leejh@pusan.ac.kr
4 Department of Information Convergence Engineering, Pusan National University, Yangsan, 50612, Republic of Korea; leejh@pusan.ac.kr
* Correspondence: jyoon@catholic.ac.kr (J.Y.)

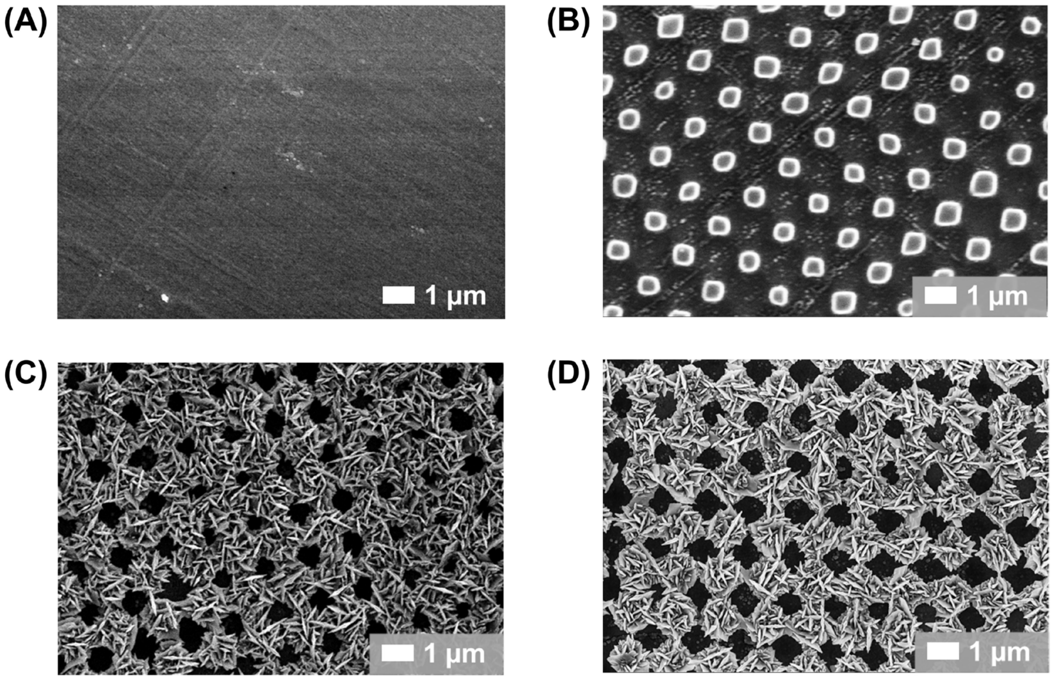

Figure S1. Zoomed-out SEM results of (A) Gold-coated flexible polymer electrode, (B) Dot-shaped PR nanopatterns, (C) FAGE, and (D) FAGE after bending.

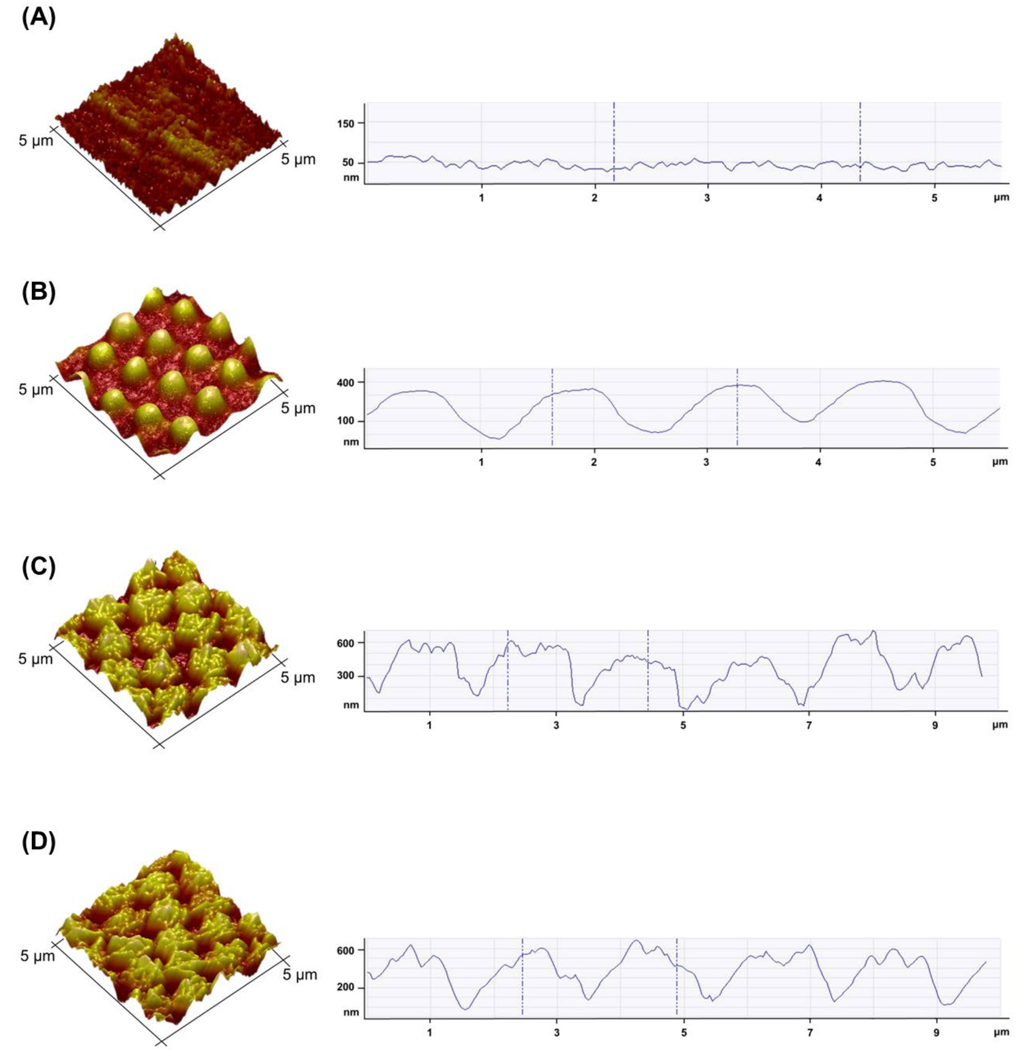

Figure S2. 3D converted AFM images with vertical investigation of (A) Gold-coated flexible polymer electrode, (B) Dot-shaped PR nanopatterns, (C) FAGE, and (D) FAGE after bending.

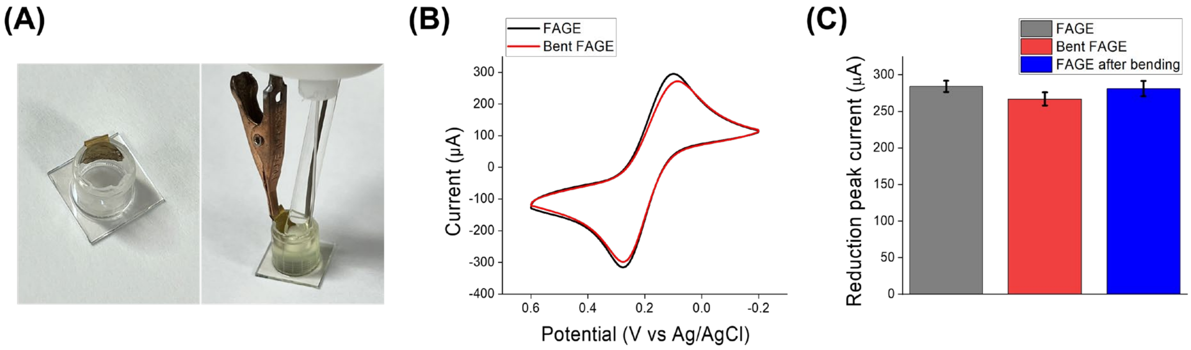

Figure S3. (A) The optical image of the bent FAGE during the electrochemical investigation, (B) Cyclic voltammograms of potassium hexacyanoferrate (II) trihydrate and hexacyanoferrate (III) on the bent FAGE, and (C) The average reduction peak current values of potassium hexacyanoferrate (II) trihydrate and hexacyanoferrate (III) on the bent FAGE, and the FAGEs before and after bending. Error bars exhibit the standard deviations of three different measurements.

DNA Sequence (5' → 3')

MIMIC-1-16	TGAAGTAGATATGGCAGCACGAGAGAATGG - SH
MIMIC-2-16	GCTCCCATA
MIMIC-1-18	ACAATATGTGCTTCTACTCAAAAAG - SH
MIMIC-2-18	TAGTAGCAC
HPV-16 Viral DNA	GTGCTGCCATATCTACTTCA
HPV-18 Viral DNA	TGTGTAGAAGCACATATTGT

Figure S4. Information of DNA sequences used in the development of flexible electrochemical biosensor composed of the MIMIC on the FAGE, and target viral nucleic acids.

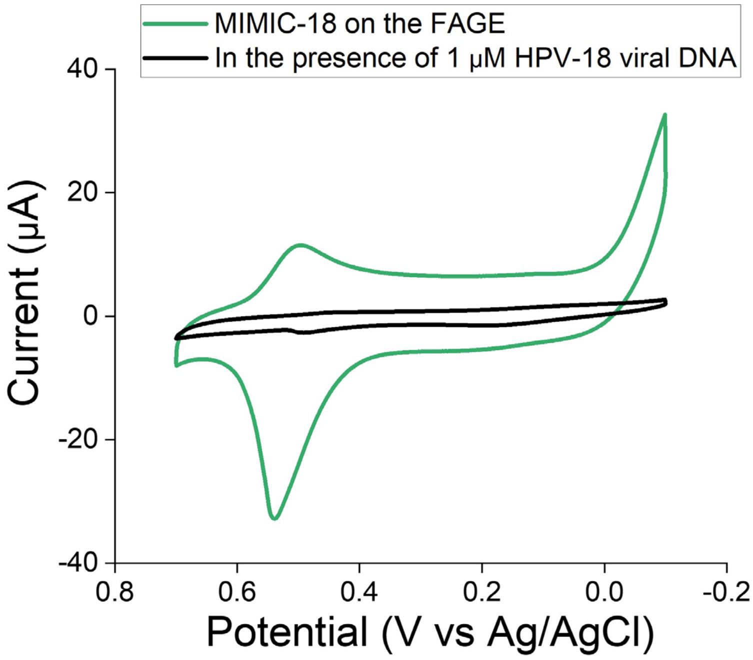

Figure S5. Cyclic voltammograms of MIMIC-18 on the FAGE and that in the presence of 1 μM HPV-18 viral DNA.

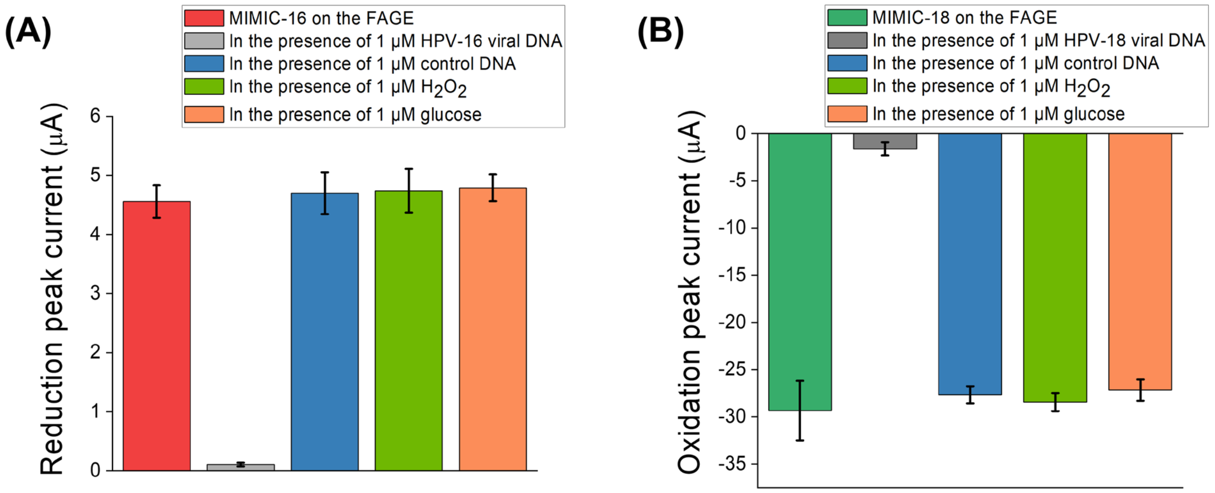

Figure S6. The average redox peak current values of the (A) MIMIC-16 and (B) MIMIC-18 during the addition of different molecules (Control DNA, hydrogen peroxide, and glucose). Error bars exhibit the standard deviations of three different measurements.
